# Railway Axle Condition Monitoring Technique Based on Wavelet Packet Transform Features and Support Vector Machines

**DOI:** 10.3390/s20123575

**Published:** 2020-06-24

**Authors:** María Jesús Gómez, Cristina Castejón, Eduardo Corral, Juan Carlos García-Prada

**Affiliations:** 1 Mechanical Department, Universidad Carlos III de Madrid (UC3M), 28982 Leganés, Spain; castejon@ing.uc3m.es (C.C.); ecorral@ing.uc3m.es (E.C.); 2 Mechanical Department, Universidad Nacional de Education a Distancia (UNED), 28040 Madrid, Spain; jcgprada@ind.uned.es

**Keywords:** railway axles, wavelet packet transform, bogie testing, condition monitoring, support vector machines

## Abstract

Railway axles are critical to the safety of railway vehicles. However, railway axle maintenance is currently based on scheduled preventive maintenance using Nondestructive Testing. The use of condition monitoring techniques would provide information about the status of the axle between periodical inspections, and it would be very valuable in the prevention of catastrophic failures. Nevertheless, in the literature, there are not many studies focusing on this area and there is a lack of experimental data. In this work, a reliable real-time condition-monitoring technique for railway axles is proposed. The technique was validated using vibration measurements obtained at the axle boxes of a full bogie installed on a rig, where four different cracked railway axles were tested. The technique is based on vibration analysis by means of the Wavelet Packet Transform (WPT) energy, combined with a Support Vector Machine (SVM) diagnosis model. In all cases, it was observed that the WPT energy of the vibration signals at the first natural frequency of the axle when the wheelset is first installed (the healthy condition) increases when a crack is artificially created. An SVM diagnosis model based on the WPT energy at this frequency demonstrates good reliability, with a false alarm rate of lower than 10% and defect detection for damage occurring in more than 6.5% of the section in more than 90% of the cases. The minimum number of wheelsets required to build a general model to avoid mounting effects, among others things, is also discussed.

## 1. Introduction

Condition monitoring aims to predict a failure in a machine before it occurs. The failure is predicted if the presence of a fault is detected. The time to a failure can be estimated with the study of faults. This allows one to stop the machine in the most convenient moment for maintenance. Condition-monitoring systems are especially necessary in critical machines, where there is a need to avoid catastrophic failures because of the potential costs and the danger posed to human lives.

The railway industry still uses scheduled preventive maintenance, with intervals based on running distance. There is no available information about the status of the machine between Nondestructive Testing inspections, which are currently the standard. Condition-monitoring solutions would constitute a valuable help for this industry in terms of providing more comfort and increasing reliability [1].

Research on condition monitoring of railway vehicles and tracks has become more substantial in recent years. Vibration signals measured at the axle box are typically used, showing that vibration signals are widely accepted for this purpose, and that the axle box is a proper location for vibration sensors in terms both of practicality and reliability. These types of signals were used for measuring railway crossing degradation in [2], for rail condition monitoring in [3], for general condition monitoring of bogies in [4], and for railway axles diagnosis in [5]. Nevertheless, these types of signals are commonly obtained with the aim of diagnosing axle bearings [6] (for a review, please see [7]) and wheels [8]. The installation of vibration sensors in the axle box provides information for the condition monitoring of many different railway elements.

Railway axles are one of the most critical elements in terms of safety. Some recent investigations studied cracked railway axle fatigue properties using experimental studies. Specifically, the work in [9] presents a fatigue crack growth rate model on the low-cycle fatigue under multiaxial loading. In [10], the authors present a fatigue crack growth model that includes the load and sequence effects and is calibrated for railway axle steels. In [11], it is highlighted that further research is required to assess the influence of a crack in the fatigue properties of a railway axle, since the crack shape is a crucial variable. In this work, several steel treatments to improve the fatigue limit are investigated.

Despite the fact that a failure in a railway axle may have catastrophic consequences, little research is to be found focusing on railway axle condition-monitoring techniques. Most of the research in this area is limited to the isolated axle (not involving the bogie), and there is a lack of experimental measurements. In [12], they use a nonlinear finite element model of a cracked railway axle to assess the use of vibration measurements for crack detection. The results show that the presence of a crack generates the harmonics of the frequency of revolution of the axle. This happens in the vertical direction and in the longitudinal direction (parallel to the rail track), and they conclude that the longitudinal direction is better for this purpose. In [13], real measurements are taken from railway axles installed in a fatigue test machine. From the Fast Fourier Transform of the acquired signals, it is concluded that the first three harmonic components of axle velocity can be used to detect cracks in railway axles, provided the damage covers more than 16% of the axle section. On the other hand, a previous related work [5] uses experimental data obtained from a full bogie set on a rig. In this work, two different wheelsets were measured at different load and speed conditions to assess the optimal conditions for crack detection.

Condition monitoring is commonly based on the control of certain features that change when a fault appears. Feature selection is not an easy task, especially when vibration signals are used, since they are complex and contain a lot of information. Signal processing tasks are required to make the available information in a raw signal easier to handle, while ensuring that the information related to the fault is retained. Different signal processing tools can be used. For the case of bearing diagnosis, Empirical Mode Decomposition (EMD), used to obtain Intrinsic Mode functions (IMFs), has been widely utilized [7]. This feature extraction technique was also used for general bogie assessment in [4]. A further development of EMD, called Complete Ensemble Empirical Mode Decomposition with Adaptive Noise (CEEMDAN), has been recently used for gear diagnosis in [14] and for feature extraction of underwater acoustic signals in [15].

However, in recent years, the Wavelet Transform (WT) has been widely used for crack diagnosis. For a review of this transform and its application for crack detection in cracked rotors, see [16]. The WT is especially suitable for this type of analysis because it works both in the time and frequency domains, which is required when dealing with faulty shaft signals where nonlinear and nonstationary effects are present. Specifically, the energy of the Wavelet Packet Transform (WPT) has been successfully used as a feature for crack detection in shafts in [17,18,19,20]. The main advantage of WPT energy is that it converts the complex structure of a raw signal into a simple energy structure in the frequency domain, notably reducing the quantity of information but conserving the information related to the crack. This feature was transferred to crack detection in railway axles in a previous work [5] with very good results. The WPT energy has also been used for other railway applications [21] to evaluate the condition of a rail fastening system. In [6], they use the WPT energy to select the frequency range that contains more fault information for axle bearing diagnosis. Later, the frequency band selected is reconstructed to the time-domain signal and Composite Multiscale Permutation Entropy (CMPE) is used as a feature.

After the feature selection stage, where features that contain information about the fault are selected, a diagnosis model must be built. Depending on the number of features and how they change with the presence of the fault, complex models can be proposed. In recent years, complex intelligent classification systems have been widely used, such as Neural Networks (NNs) for axles in [5,19], Support Vector Machines (SVMs) for predicting rail track degradation in [14,22], and to diagnose axle bearings in [23].

The combination of WPT with SVMs is a proven solution for fault identification that has been used for structural health monitoring in [24], for general rotating machinery in [25], for gears in [26], and for bearings in [27,28].

The present work details a method for real-time condition monitoring of railway axles. Experimental vibration signals obtained at the axle box of a full bogie installed on a rig were obtained. Four different wheelsets were tested, each one with four different crack levels. The WPT energy of the signals was calculated and the feature selection was done, thus, a frequency region that seems to contain reliable information about the crack was identified. The information of the features selected in the current state (for diagnosis) and the healthy state (established as a reference) were used to build a reliable SVM diagnosis model. The minimum number of tested wheelsets required to build a general model is also discussed in this work.

## 2. Methodology

After the literature review, the signal processing tool was selected. Among the wide variety of possibilities, the WPT energy was chosen for the crack diagnosis in railway axles due to its proven effectiveness at diagnosing cracked shafts in other applications, as detailed in the previous section. The main advantage of this technique is that it is especially suitable for work with nonstationary signals and transient effects, and after the parameters are chosen, simple patterns that keep information related to the fault are obtained in a straightforward way.

Once the WPT parameters were selected (detailed below), the acquired vibration signals were processed to calculate the WPT energy. For the feature selection stage, the trends in the WPT energy as the defect began to grow were studied first. A packet (which represents a frequency region) was selected as a feature when its WPT energy showed a clear trend with respect to the crack size, because the packet is considered to have relevant information about the crack condition.

To build a diagnosis model able to automatize the diagnosis of the status of the axle, a classification system was used. The aim was to use a classification system that maximizes the success rate for the features selected. Thus, the features selected were used to train several classification system types such as NNs, decision trees, discriminant analysis, logistic regression, nearest neighbors, naive Bayes, ensemble classification, and SVMs. After benchmarking, the SVM were selected.

### 2.1. WPT Energy

The WPT is a time-frequency domain signal processing tool that divides the signal into a certain number of packets of the same frequency resolution.

**Figure 1:**
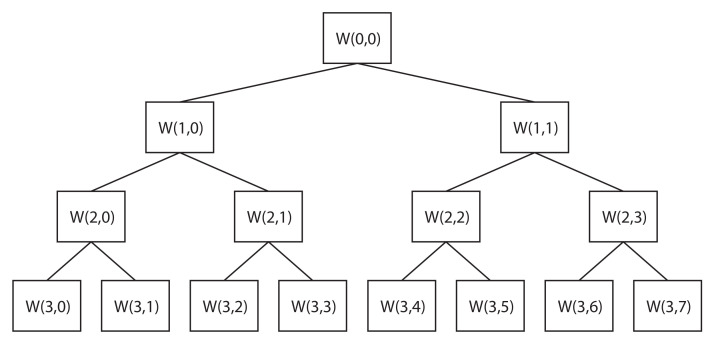
Wavelet Packet Transform (WPT) decomposition procedure until decomposition level 3 [20].

The WPT transform calculates the correlation coefficients between the analyzed signal and the wavelet function, by means of digital filters. The signal is decomposed by dividing the spectrum into separate halves with a low-pass filter and a high-pass filter [29]. At each new decomposition, the coefficients of the previous iteration are assumed [30]. Figure 1 shows the scheme for a WPT decomposition level 3, where 8 packets of the same frequency are obtained, where W(k,j) represents the correlation coefficients and *k* is the decomposition level. The parameter *j* represents the values of the position of the packet within its decomposition level, which, in this work, are ordered according to the natural order of frequency ranges in which the signal is divided.

The correlation coefficients W(k,j) have the structure of Equation (1):

(1) $$W\left(k,j\right)=\left\{w_{1}\left(k,j\right),\ldots ,w_{N}\left(k,j\right)\right\}=\left\{w_{i}\left(k,j\right)\right\},$$

where *i* is the position of the coefficient within its packet.

The decomposition level determines the number of packets obtained. The frequency resolution of the signal is equally distributed in the packets obtained, therefore, when the number of packets increases, the frequency resolution of each packet is lower. Using a decomposition level *k*, the number of packets obtained is $2^{k}$. Taking into account the global frequency resolution $F_{r}$ (half of the sampling frequency, $F_{s}$, according to the Nyquist theorem), the frequency resolution $f_{r}$ of each packet is given by Equation (2) [31].

(2) $$f_{r}=\frac{F_{r}}{2^{k}}=\frac{F_{s}}{2\cdot 2^{k}}.$$

The absolute energy of each WPT packet is calculated as the sum of all squares of the coefficients of each packet, according to Equation (3):

(3) $$E_{k,j}=\sum_{i}{\left\{w_{i}\left(k,j\right)\right\}}^{2}.$$

**Figure 2:**
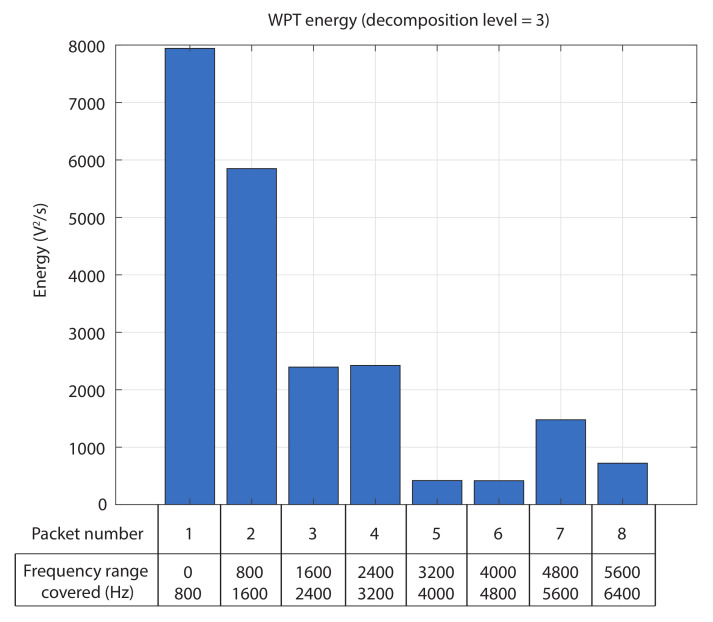
WPT energy of a signal acquired with a sampling frequency $F_{s}=12.8$ kHz, using db6 mother wavelet and decomposition level 3 (8 packets obtained).

Figure 2 shows an example of the WPT energy decomposition of a signal acquired with $F_{s}=12.8$ kHz, using Daubechies 6 (db6) mother wavelet and decomposition level 3, where the signal is decomposed into 8 packets or 8 WPT energy values. The frequency range covered by each packet is also shown.

To calculate the WPT energy, the wavelet function and the decomposition level must be selected. In this work, the Daubechies 6 wavelet function was used in line with the previous work, such as [5,17,18,20], where this function was applied with very good results.

Regarding the decomposition level, the level k=6 is selected after a sensitivity analysis—considering the frequency resolution of each packet and the success rates. Table 1 shows the frequency parameters obtained for this value.

### 2.2. Support Vector Machines

SVMs are intelligent classification systems that classify the features received at the input in two possible different classes. An SVM model classifies data by calculating the hyperplane that best separates the data from the two classes, maximizing the margin between them. In cases where the data from the two classes are not separable by a hyperplane, the established hyperplane may separate some, but not all data features.

An SVM classification model is created using a supervised learning algorithm, where features with known classes are used to train the model. For the training of an SVM model, an optimization problem is solved to find the decision boundary (hyperplane) to classify the features. Let $\left(x_{i},y_{i}\right)$ be the training dataset, where $x_{i}$ ϵ $R^{D}$ is the feature vector (D is the dimension), and $y_{i}=\left\{-1,1\right\}$ are the two possible classes. The dataset has N samples with i=1,2,…,N. The equation of the hyperplane is shown in Equation (4).

(4) $$f\left(x\right)=x^{\prime }\beta +b=0,$$

where β ϵ $R^{D}$ and *b* is a real number. This hyperplane is obtained by finding the β and *b* that minimize ∥β∥ such that for all data points $\left(x_{i},y_{i}\right)$, $y_{i}\cdot f\left(x_{i}\right)\geq 1$. This can be solved using a quadratic optimization problem. The optimization problem can be formulated by applying Lagrangian multipliers α, thus maximizing the objective function shown in Equation (5) [32].

(5) $$\max_{\alpha }L_{D}=\sum_{i=1}^{N}\alpha_{i}-\frac{1}{2}\sum_{i}^{N}\sum_{j}^{N}\alpha_{i}\alpha_{j}y_{i}y_{j}K\left(x_{i},x_{j}\right),$$

where $\alpha_{i}$ and $\alpha_{j}$ are Lagrangian multipliers, and $x_{i}$ and $x_{j}$ are two training vectors. The restrictions of the optimization problem are in Equations (6) and (7).

(6) $$\sum_{i}^{N}\alpha_{i}y_{i}=0,$$

(7) $$0\leq \alpha_{i}\leq C,$$

where *C* is the penalty parameter that determines the tolerance to misclassification errors.

Regarding the kernel function $K(x_{i},x_{j})$, it can be a linear, Gaussian, polynomial, or sigmoid function. In this work, a linear function is used because by being more simple it offered better results. The linear function is shown in Equation (8) [32].

(8) $$K\left(x_{i},x_{j}\right)=x_{i}^{\prime }x_{j}.$$

Once the model is trained, it is cross-validated, and then it can be used to predict the class of new data. SVMs are among the most widely used classification systems due to their high classification accuracy and good generalization performance, even with few samples [22,33,34].

### 2.3. Proposed Fault Diagnosis Method

The fault diagnosis method proposed in this work for railway axles is as follows:

(1) Perform WPT energy calculations using Daubechies 6 mother wavelet on the vibration signals and decompose them into 64 packets (frequency bands);
(2) Represent the mean energy of each packet for each crack size tested versus the crack size. Those packets that experience changes in the crack sizes will be chosen as features;
(3) Input the feature vectors selected to train several classification system types trough supervised learning. All available data are used to check the diagnosis model that best fits the data. All the models trained are cross-validated by partitioning the dataset into 10 disjoint folds. For each fold, a model is trained using the out-of-fold data, and the accuracy of each model using the in-fold data is calculated. The average accuracy of the 10 models is used to estimate the predictive accuracy of the final model trained with all the data;
(4) The classification system that gives the best success rate is chosen. If similar success rates are obtained, the decision is made according to the classifier characteristics and simplicity. In this application, protection against overfitting and high-accuracy are critical;
(5) A sensitivity analysis to check the influence of the number of wheelsets used to build the model is performed.

**Figure 3:**
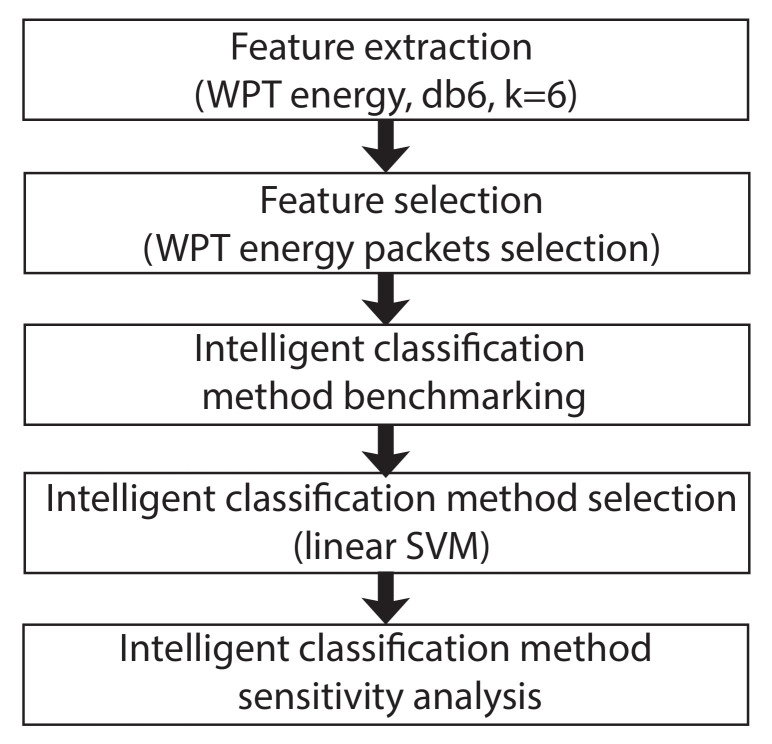
Overall methodology flow chart.

Figure 3 shows the flowchart of the methodology described.

## 3. Experimental Setup

In the present section, the axle, the test rig, the tests performed, and the measurement chain are described.

### 3.1. Axle and Cracks Description

**Figure 4:**
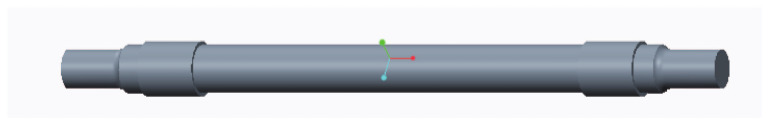
Axle model for modal analysis.

The axle tested has a diameter of 130 mm and a total length of 2.35 m. The CAD (Computer Aided Design) model of the axle is shown in Figure 4 and was used to perform a modal analysis using the finite element method. The natural frequencies were obtained and the first five are shown in Table 2.

Axles with several crack conditions were tested. The first test was carried out when the axle was healthy. Later, cracks were induced, using a cutting tool, in the middle section of the axle. Cracks were created with the wheelset installed to avoid mounting effects. Thus, any changes observed in the signals measured for each wheelset could only be attributed to the appearance of the defect. Three different crack levels were cut and they were measured in terms of depth (mm) and damaged area percentage with respect to the cross section of the axle. These values are defined in Table 3.

**Figure 5:**
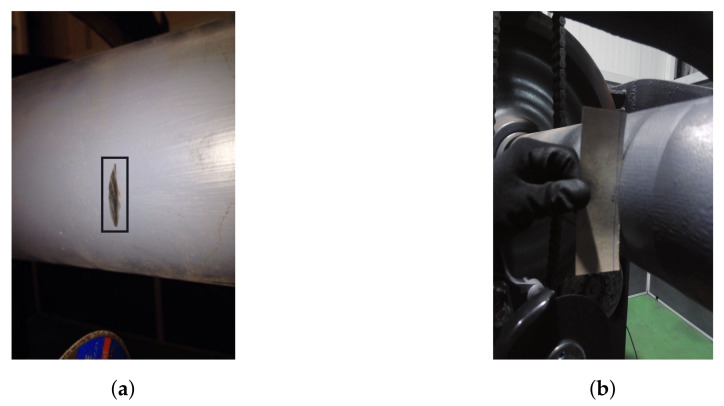
Defect level D1: (**a**) surface detail; (**b**) depth detail.

Figure 5a shows the surface appearance of a D1 level crack in the middle section of the axle. The depth of a D1 level crack can be observed in Figure 5b, where a sheet is inserted into it.

### 3.2. Test Rig

A wheelset comprises the axle, two wheels, and brakes. The bogie is the structure in which the wheelsets and the suspension system are installed in railway vehicles. The bogie is connected to the wheelset by means of the axle using the axle boxes, where the axle bearings are set.

**Figure 6:**
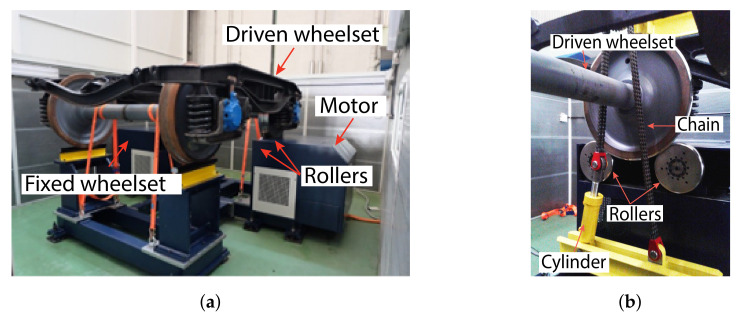
The test rig showing (**a**) bogie Y21 Cse set on the rig (without loading system) and (**b**) driven wheelset supported on the rollers and loading system with hydraulic cylinder and chain.

The vibration response of four different wheelsets—WS1, WS2, WS3, and WS4, working under a constant load in a steady state—was measured. The tested wheelsets are installed in the bogie Y21 Cse, which is typically used as a trailer for freight transport. The bogie is supported by two wheelsets and the whole system is set on a rig specifically designed by Dannobat Railway Systems to test bogies, as shown in Figure 6a.

The rig comprises a driving system that actuates one of the driven wheelsets using rollers. Vibration sensors are installed on both axle boxes of the driven wheelset. The other wheelset stands on the fixed bench. The rig has hydraulic actuators that apply a constant vertical load to the bogie. The loading system pushes a beam against the bogie using a chain, as shown in Figure 6b.

### 3.3. Test Conditions

Tests were performed under a constant load in a steady state after a running-in for each crack level. Measurements were taken in the two possible rotation senses, clockwise (cw) and counterclockwise (ccw), for all cases. The tests conditions are defined in Table 4.

### 3.4. Acquisition System

**Figure 7:**
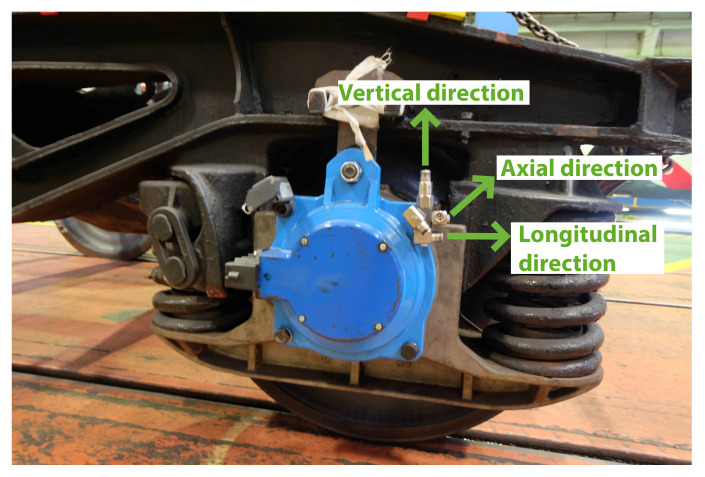
Sensors installed in the axle box cover and measurement directions.

Six uniaxial acceleration sensors were installed (model CMSS-RAIL-9100) by SKF (Gothenburg, Sweeden) in each tested wheelset. Three of them were set on each axle box cover (three on the right-hand side (RHS) and three on the left-hand side (LHS)) with the same orientations on both sides. Two of them were oriented to measure vibration in radial directions—specifically, the vertical and the longitudinal (parallel to the rail track) directions—and the third was oriented to measure the axial direction vibration. The installed sensors can be seen in Figure 7.

The parameters of each measured signal are detailed in Table 5.

Each test lasted around 30 min, enough time to acquire a minimum number of 60 signals. Finally, for each crack level, the number of available signals was around 960 for each vibration direction, corresponding to the four wheelsets measured.

## 4. Results and Discussion

In this section, firstly, the results of the features selection using the WPT energy trends and the benchmarking for the different classification systems tested are discussed. Then, the linear SVM model results and the sensitivity analysis are detailed and discussed.

### 4.1. Feature Extraction and Selection Using WPT

The evolution of the WPT energy of the 64 packets versus the crack sizes was studied for the four wheelsets in the three acceleration directions. There were some packets where the WPT energy showed an upward trend when the crack appears and grows. This trend was more obvious in the radial directions (vertical and longitudinal) than in the axial direction. More specifically, packet number 2 (100–200 Hz) demonstrated this behavior for all the wheelsets, in both rotation senses, and for all the sensors on both sides in the radial directions. The WPT energy of packet number 2 increased with the crack size with a RHS–LHS and ccw–cw symmetry for the four wheelsets tested, both for the vertical and longitudinal directions. This repeatability was not observed in the other packets. Thus, the WPT energy of packet number 2 gives reliable information about the crack condition. Therefore, to build a general model, the WPT energy of packet number 2 was selected as feature.

Packet number 2 (100–200 Hz) includes the first natural frequency of the axle: 142.37 Hz. This is consistent with previous work, such as [20], where it is confirmed that the energy related to the first natural frequency in the radial directions is affected by the crack.

**Figure 8:**
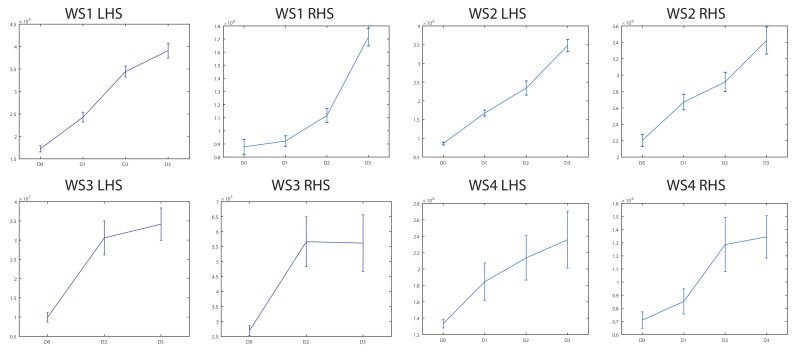
Evolution of energies (mean and standard deviation; (V${}^{2}$/s)) versus crack levels D0, D1, D2, and D3 for packet number 2 in longitudinal acceleration.

Figure 8 shows the evolution of energies versus the crack size for packet number 2 for acceleration in the longitudinal direction. For the case of WS3, there are no available data for the D1 level crack due to a connection problem during the acquisition.

As can be observed in Figure 8, the energy values of packet 2 increased when the crack appeared as compared with the energy obtained in the healthy condition. However, the mean energy value in the healthy condition is very different for each case. This led us to conclude that it would be necessary to use an estimator for the energy value of the healthy condition to diagnose the condition of the axle. Then, the WPT mean energy of the healthy axle was included as an input for the classification system.

### 4.2. Intelligent Classification Systems Benchmarking

The dimension of the input vector is two, i.e., $x_{i}=\left\{x1_{i},x2_{i}\right\}$ for i=1,2,…N, where N=3555. The feature x1 represents the mean value of the WPT energy of packet number 2 in the healthy condition for each case, and x2 is the WPT energy value of packet number 2. All WPT energies were calculated using the vibration signals measured in the longitudinal direction. The possible outputs (predicted classes) are $y_{i}=\left\{-1,1\right\}$, which represent the healthy and cracked conditions, respectively.

All features corresponding to WS1, WS2, WS3, and WS4 were used for the supervised training, thus, full diagnosis models were built using different intelligent classification system types. Each model was validated using cross-validation with 10 folds. For each classification system, the dataset was divided into 10 disjoint folds and 10 different fits were performed. The accuracy was calculated as the average accuracy of the 10 models trained for each case. Table 6 shows the accuracy for all the classification systems tested, where it can be seen that the linear SVM model offers the best results. Other classification systems offer similar results, but the linear SVM model was chosen because of its high accuracy and protection against overfitting.

### 4.3. Linear SVM Model

**Figure 9:**
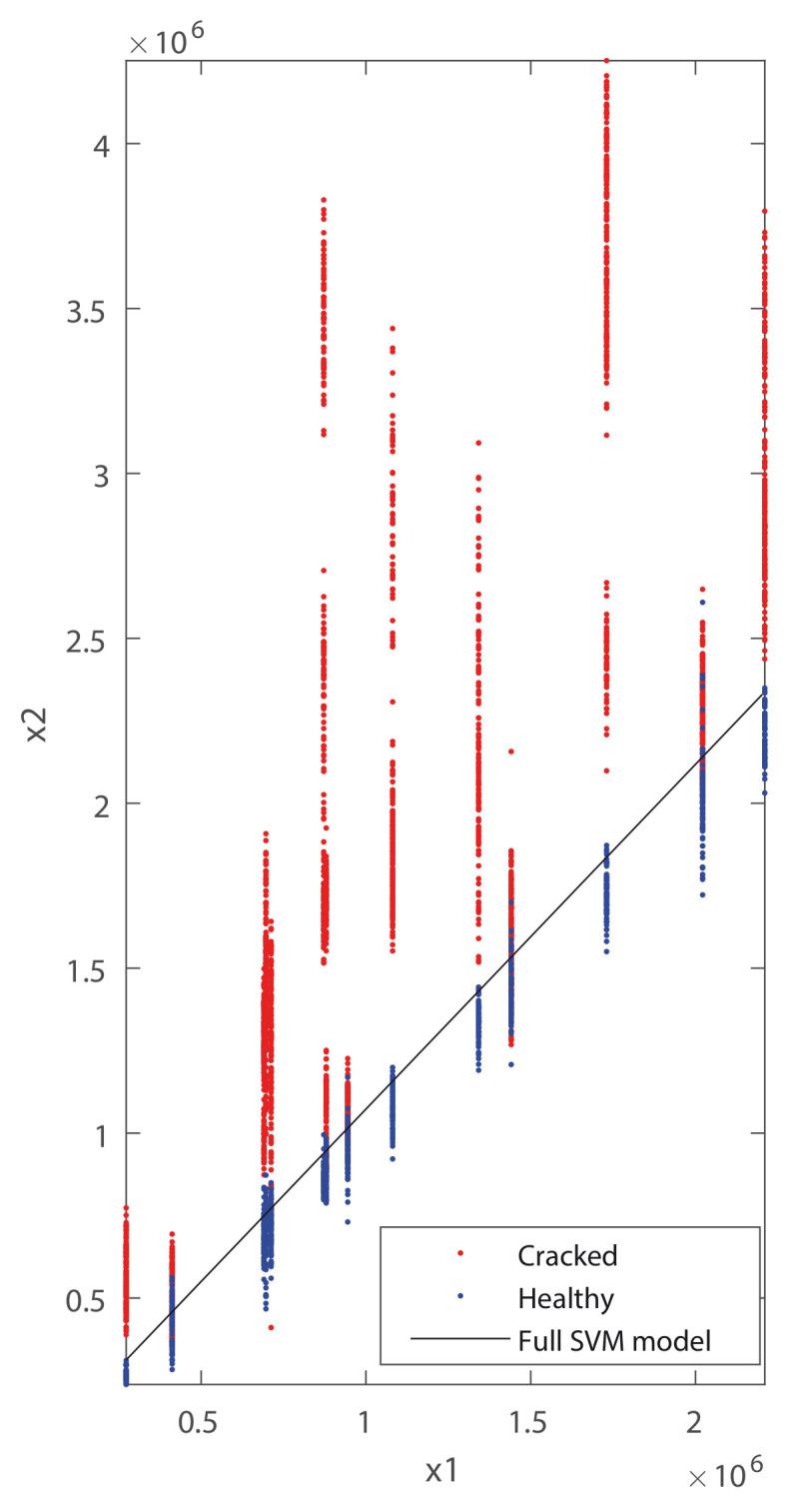
x1 (V${}^{2}$/s) vs. x2 in (V${}^{2}$/s) for wheelset 1 (WS1), WS2, WS3, and WS4; the healthy condition is represented in blue and the cracked condition in red. The line represents the full linear SVM model that best separates the data corresponding to the healthy and cracked axle.

Figure 9 shows a representation of x1 versus x2 for all the available data from the four wheelsets, with the full linear SVM model that best separates the data of the healthy condition from the data of the cracked condition.

The global success rate of the full linear SVM model is 88.98% (calculated as the percentage of features that are correctly classified among all available data).

The results obtained for the full linear SVM model, which uses features calculated from WS1, WS2, WS3, and WS4, are very promising. However, the question is how many wheelsets are necessary to build a reliable model able to predict the data from new wheelsets that have not been used to train it, i.e., how many wheelsets are required to build a general model?

**Figure 10:**
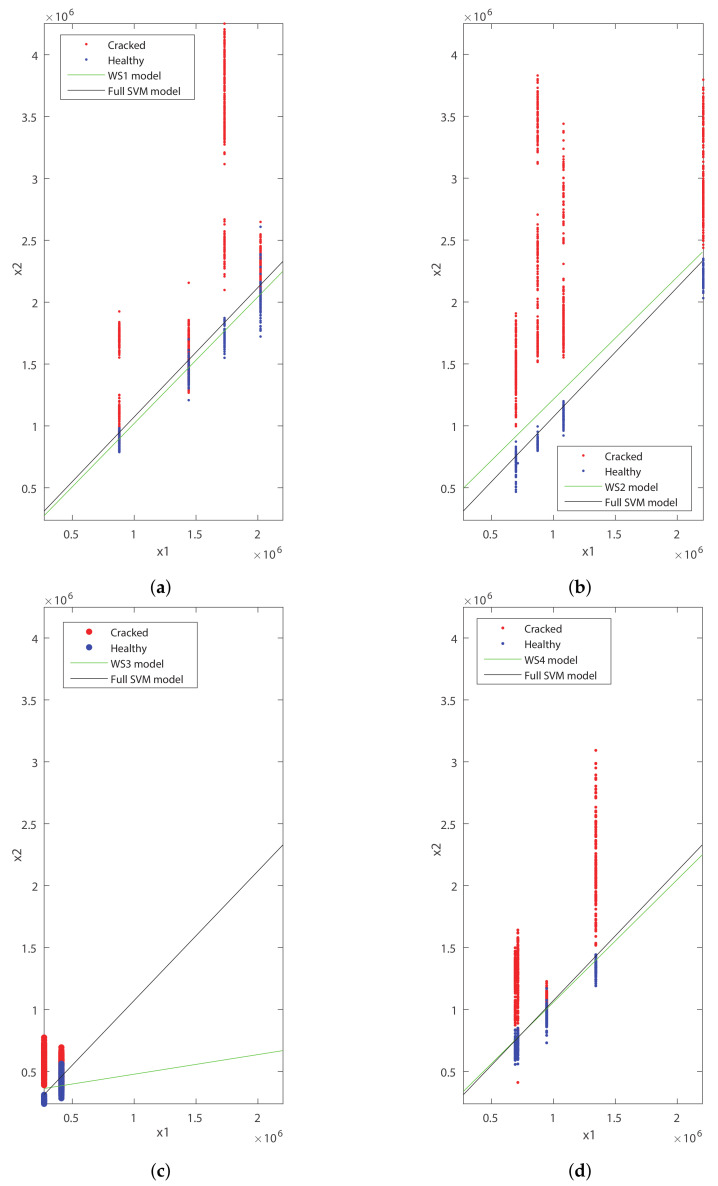
Full linear Support Vector Machines (SVM) model and x1 (V${}^{2}$/s) vs. x2 in (V${}^{2}$/s) for (**a**) the WS1 features and WS1 model; (**b**) the WS2 features and WS2 model; (**c**) the WS3 features and WS3 model; and (**d**) the WS4 features and WS4 model.

For this purpose, a sensitivity analysis to check the influence of the number of wheelsets used to build the model was performed. Firstly, the four possible linear SVM models trained with the data from one unique wheelset were trained, establishing the line that best separates the data from the cracked and healthy condition for each wheelset. Figure 10 shows the representation of the features x1 versus x2 and the linear SVM model built for each wheelset.

As can be seen in Figure 10, a model trained with data from a unique wheelset may differ a lot from the full linear SVM model, which is more general. The model built for WS3 is very different from the full linear SVM model. This leads us to conclude that the use of a unique wheelset to create the model may be conducive to bad diagnosis results.

All the possible combinations of the features of one, two, and three wheelsets were used to train linear SVM models. Each model was tested at classifying the data from the rest of the wheelsets that had not been used for training (the testing data). The success rate was calculated as the percentage of the testing data that were correctly classified. The trained models and the obtained results are shown in Table 7.

As can be seen in Table 7, when the features of only one wheelset are used to train the linear SVM model, the success rates are between a 72.05% and 88.68%. However, when the features of two wheelsets are used, the results are between 80.07% and 91.28%. When the data from three wheelsets are used for training, the results are between 80.19% and 95.10%. This leads us to conclude that when the number of wheelsets used to train the classification system increases, the accuracy of the model improves. This means that the diagnosis model is more general and the classification of the data from new wheelsets that have not been used for training is more reliable.

**Figure 11:**
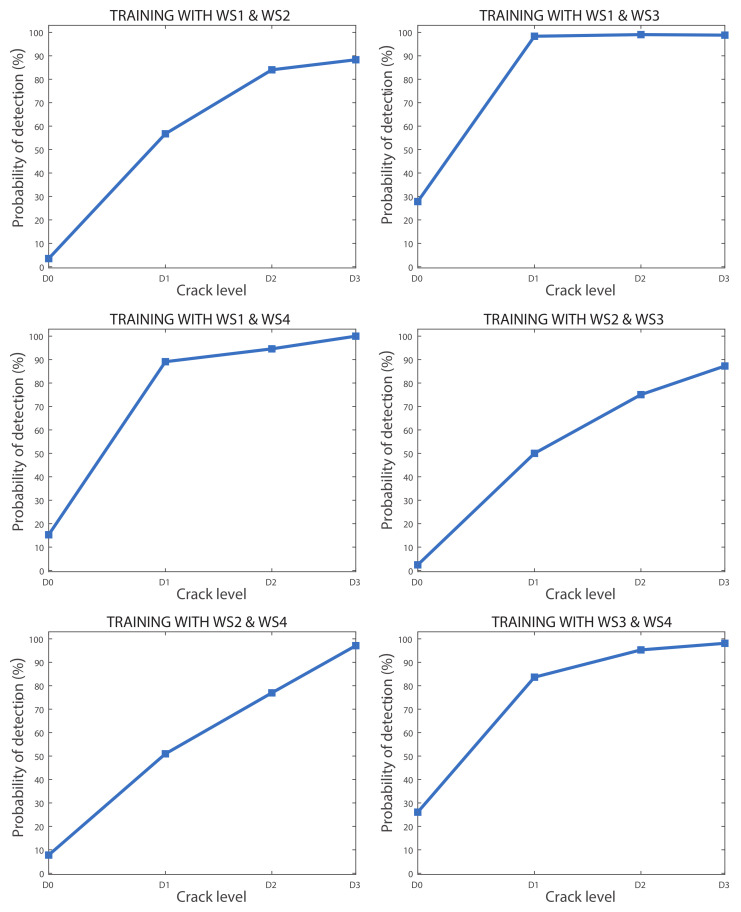
Probability of detecting a crack (%) for each crack size for all models where the data from two wheelsets were used for training.

The separated success rates for each crack level were calculated for the linear SVM models where two wheelsets were used for training. Figure 11 shows the probability that each of the six models would affirm that a defect exists depending on the crack level. The results show that, despite the defects being detected with a high reliability, there is a high risk of false alarms, which reaches almost 30% in two of the six models.

**Figure 12:**
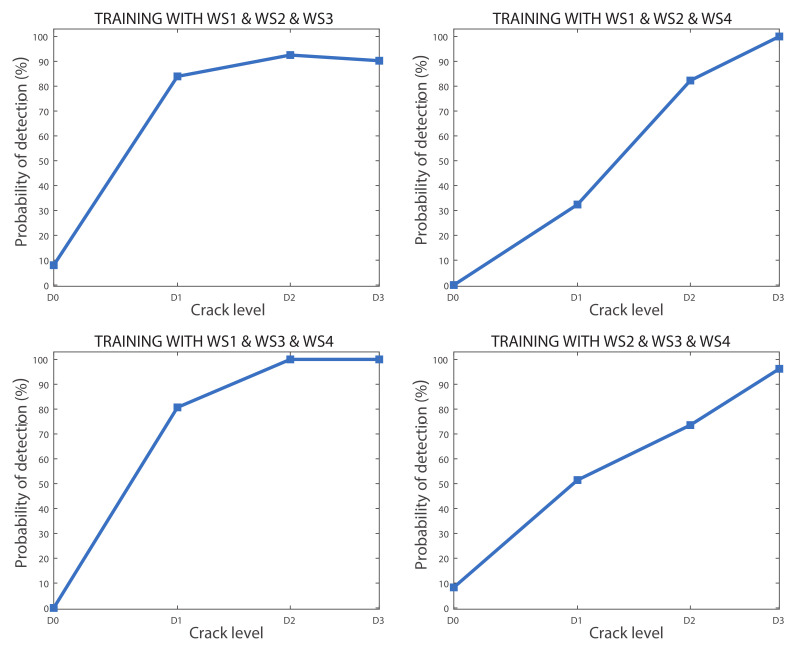
Probability of detecting a crack (%) for each crack size for all models where the data from three wheelsets were used for training.

The separated success rates for each crack level were also calculated for the linear SVM models where the data from three wheelsets were used for training. Figure 12 shows the probability of each model affirming that a defect exists depending on the crack level. In this case, the false alarm rate is 10% in the most adverse scenario. On the other hand, axles with a D1 level crack were not well classified, but the D2 level defect was detected with 70% reliability in the worst case, and the D3 level defect was detected with high reliability, reaching 90% in the worst case scenario.

### 4.4. Discussion

These results should be taken into account when considering how to build a diagnosis model for condition monitoring of a railway axle.

It must be considered that as the number of wheelsets used to build the model increases, the model is more general and the predictions are better. In this case, the available data show that a model built with three wheelsets can detect a crack of 15 mm (6.5% of the damaged section) in more than 90% of cases, with a false alarm rate lower than 10% in a new wheelset that had not been used for training. If a higher reliability is required, the number of wheelsets used to build the model should be increased. Further research is needed to establish the maximum reliability of this technique, and for how many wheelsets this reliability can be maintained.

**Figure 13:**
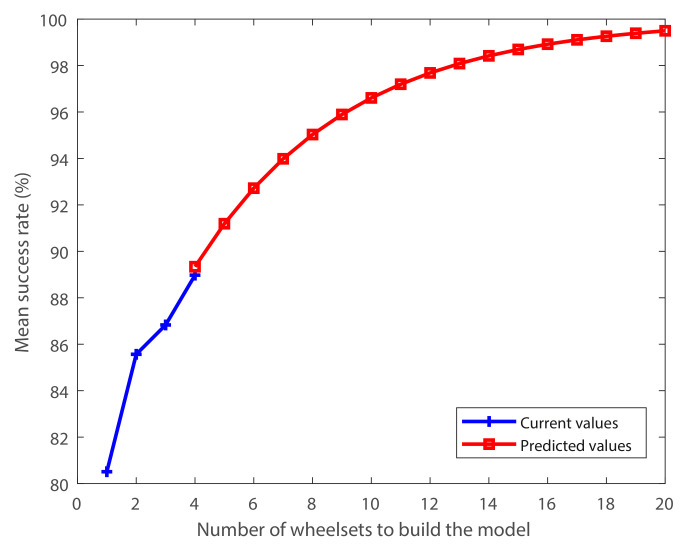
Current and predicted mean success rates for the models as a function of the number of wheelsets used to build the model.

However, the mean success rates of the available models versus the number of wheelsets used to build each model were adjusted to an exponential function with an R-square of 0.95. This function is shown in Figure 13.

According to authors experience, a reference value of the healthy condition must always be used as an input for the model for correct accuracy. This is due to the differences in manufacturing, mounting, and/or environmental effects of the mechanical system, which can cause the vibration signature of each wheelset to differ greatly despite the wheelset model being the same.

Building these types of diagnosis models may involve substantial effort in reality; however, the benefits can also be very valuable. Nowadays, railway axles are generally inspected periodically using nondestructive testing, mainly ultrasound testing, involving complete dismounting and downtime of the units. Furthermore, there is no information available about the status of the axle between inspections. This type of technique, which of course needs to be tested in real conditions, could be a valuable complement to the current periodical inspections in the future.

## 5. Conclusions

The present work describes a vibration-based technique for railway axle condition monitoring. The technique was tested on vibration signals taken from a rig where a full bogie, model Y21, was set. Different tests were performed for four different wheelsets under four different transversal crack depths in the axle, from healthy to 15 mm. The acquired signals were processed by means of WPT energy. The results show that if a healthy axle produces a certain level of vibration at its first natural frequency, a crack in the axle causes a significant increase in this vibration level. This energy increase, as compared to the healthy condition, can be used to build a diagnosis model to predict the condition of future wheelsets of the same type. Several types of classification system were tested, and a linear SVM seemed to be the best option.

The full linear SVM diagnosis model, using data from all the tested wheelsets for training, seems to work properly. However, a sensitivity study to evaluate the generalization capability of the model was done. If data from only one wheelset were used to build the linear SVM model, the classification of the data from new wheelsets that had not been used to build it was not accurate. When two wheelsets were used, the success rates were higher; however, the number of false alarms and the capability of detecting cracks were not reliable enough. The best results were obtained when a linear SVM model, using data from three wheelsets, was used. In all possible combinations where a three-wheelset model was used to predict the data from a new wheelset that had not been used for training, the false alarm rate was under 10% and D3 level cracks (for damage occurring in more than 6.5% of the section) were detected in more than 90% of the cases. If a higher reliability is required, the diagnosis model must be built using data from more wheelsets. These results are extremely valuable since they were obtained from a real bogie, and they show that this technology may have a future in the railway industry.

## Figures and Tables

**Table 1:** Decomposition level and frequency parameters.

Decomposition Level *k*	6
Number of packets $2^{k}$	64
Sampling frequency $F_{s}$ (kHz)	12.8
Signal frequency resolution $F_{r}$ (kHz)	6.4
Frequency resolution of each packet $f_{r}$ (Hz)	100

**Table 2:** Natural frequencies.

Mode	1	2	3	4	5
**Frequency (Hz)**	142.37	387.42	692.28	733.68	1121.73

**Table 3:** Crack levels and depths.

Crack Level	Depth (mm)	Damaged Section (%)
**D0**	0	0
**D1**	5.7	1.5
**D2**	10.9	4
**D3**	15	6.5

**Table 4:** Tests conditions.

**Load Value (t)**	10
**Rotation Speed (Hz)**	30
**Equivalent Train Speed (km/h)**	50
**Rotation Senses**	cw and ccw

**Table 5:** Parameters of signals measured for each signal.

**Sampling Frequency** $F_{s}$	12.8 kHz
**Acquisition Time (s)**	1.28
**Number of Points (N)**	16,384 $\left(2^{14}\right)$

**Table 6:** Parameters of signals measured for each signal.

Classification System Type	Classification System	Accuracy (%)
NN	RBF	87.7
Decision Tree	Tree	85.2
Discriminant Analysis	Linear discriminant	76.4
	Quadratic discriminant	86.7
Logistic regression	Logistic regression	88.7
Support Vector Machine	Linear SVM	88.7
	Quadratic SVM	68.9
	Cubic SVM	58.7
	Gaussian SVM	87.9
K nearest neighbors	KNN	88.2
	Cosine KNN	82.7
	Cubic KNN	88.7
	Weighted KNN	88.6
Ensemble	Boosted trees	88.4
	Bagged trees	87.6
	Subspace discriminant	73.2
	Subspace KNN	53.5

**Table 7:** Packets selected as the best features and the related frequency bands.

Number of Wheelsets for Training	Training Data	Testing Data	Success Rate (%)
1	WS1	WS2, WS3, WS4	88.68
	WS2	WS1, WS3, WS4	72.05
	WS3	WS1, WS2, WS4	73.11
	WS4	WS1, WS2, WS3	88.20
2	WS1, WS2	WS3, WS4	84.32
	WS1, WS3	WS2, WS4	91.28
	WS1, WS4	WS2, WS3	90.15
	WS2, WS3	WS1, WS4	80.07
	WS2, WS4	WS1, WS3	80.34
	WS3, WS4	WS1, WS2	87.24
3	WS1, WS2, WS3	WS4	90.30
	WS1, WS2, WS4	WS3	81.73
	WS2, WS3, WS4	WS1	80.19
	WS1, WS3, WS4	WS2	95.10
